# Regional lung aeration and ventilation during pressure support and biphasic positive airway pressure ventilation in experimental lung injury

**DOI:** 10.1186/cc8912

**Published:** 2010-03-16

**Authors:** Marcelo Gama de Abreu, Maximiliano Cuevas, Peter M Spieth, Alysson R Carvalho, Volker Hietschold, Christian Stroszczynski, Bärbel Wiedemann, Thea Koch, Paolo Pelosi, Edmund Koch

**Affiliations:** 1 Pulmonary Engineering Group, Department of Anaesthesiology and Intensive Care Therapy, University Hospital Carl Gustav Carus, Technical University of Dresden, Fetscherstr. 74, 01307 Dresden, Germany; 2 Department of Anaesthesiology and Intensive Care Therapy, University Hospital Carl Gustav Carus, Technical University of Dresden, Fetscherstr. 74, 01307 Dresden, Germany; 3 Institute of Radiology, University Hospital Carl Gustav Carus, Technical University of Dresden, Fetscherstr. 74, 01307 Dresden, Germany; 4 Institute of Medical Informatics and Biometry, Medical Faculty Carl Gustav Carus, Technical University of Dresden, Löscherstr. 18, 01309 Dresden, Germany; 5 Department of Ambient, Health and Safety, University of Insubria, Servizio di Anestesia B, Ospedale di Circolo e Fondazione Macchi viale Borri 57, 21100 Varese, Italy; 6 Clinical Sensoring and Monitoring, Department of Anaesthesiology and Intensive Care Therapy, University Hospital Carl Gustav Carus, Technical University of Dresden, Fetscherstr. 74, 01307 Dresden, Germany

## Abstract

**Introduction:**

There is an increasing interest in biphasic positive airway pressure with spontaneous breathing (BIPAP+SB_mean_), which is a combination of time-cycled controlled breaths at two levels of continuous positive airway pressure (BIPAP+SB_controlled_) and non-assisted spontaneous breathing (BIPAP+SB_spont_), in the early phase of acute lung injury (ALI). However, pressure support ventilation (PSV) remains the most commonly used mode of assisted ventilation. To date, the effects of BIPAP+SB_mean _and PSV on regional lung aeration and ventilation during ALI are only poorly defined.

**Methods:**

In 10 anesthetized juvenile pigs, ALI was induced by surfactant depletion. BIPAP+SB_mean _and PSV were performed in a random sequence (1 h each) at comparable mean airway pressures and minute volumes. Gas exchange, hemodynamics, and inspiratory effort were determined and dynamic computed tomography scans obtained. Aeration and ventilation were calculated in four zones along the ventral-dorsal axis at lung apex, hilum and base.

**Results:**

Compared to PSV, BIPAP+SB_mean _resulted in: 1) lower mean tidal volume, comparable oxygenation and hemodynamics, and increased PaCO_2 _and inspiratory effort; 2) less nonaerated areas at end-expiration; 3) decreased tidal hyperaeration and re-aeration; 4) similar distributions of ventilation. During BIPAP+SB_mean_: i) BIPAP+SB_spont _had lower tidal volumes and higher rates than BIPAP+SB_controlled_; ii) BIPAP+SB_spont _and BIPAP+SB_controlled _had similar distributions of ventilation and aeration; iii) BIPAP+SB_controlled _resulted in increased tidal re-aeration and hyperareation, compared to PSV. BIPAP+SB_spont _showed an opposite pattern.

**Conclusions:**

In this model of ALI, the reduction of tidal re-aeration and hyperaeration during BIPAP+SB_mean _compared to PSV is not due to decreased nonaerated areas at end-expiration or different distribution of ventilation, but to lower tidal volumes during BIPAP+SB_spont_. The ratio between spontaneous to controlled breaths seems to play a pivotal role in reducing tidal re-aeration and hyperaeration during BIPAP+SB_mean_.

## Introduction

Maintenance of spontaneous breathing activity during ventilatory support in acute lung injury (ALI) may improve pulmonary gas exchange, systemic blood flow, and oxygen supply to the tissues [1]. Most importantly, spontaneous breathing activity may contribute to decrease the time of ventilatory support and the length of stay in the intensive care unit [2]. Although pressure support ventilation (PSV) is the most frequently used form of assisted mechanical ventilation [3], there is increasing interest in biphasic positive airway pressure with superposed spontaneous breathing (BIPAP+SB_mean_) [4]. PSV is a pressure-limited, flow-cycled mode in which every breath is supported by a constant level of pressure at the airways, thus the tidal volume (V_T_) and inspiratory flow may adapt to the demands of the patient [5]. In contrast, BIPAP+SB_mean _is a combination of time-cycled controlled breaths at two levels of continuous positive airway pressure (BIPAP+SB_controlled_) and non-assisted spontaneous breathing (BIPAP+SB_spont_) [4]. Compared with controlled mechanical ventilation and PSV, a possible advantage of non-assisted spontaneous breath during BIPAP+SB_mean _is that they may generate higher transpulmonary pressures in dependent lung areas, contributing to lung recruitment, reduction of cyclic collapse/reopening and improvement of ventilation/perfusion matching [6-8].

Previous studies comparing PSV with BIPAP+SB_mean _have not assessed the distribution of both aeration and ventilation [6,9,10]. In experimental ALI, we observed that aeration compartments of the whole lungs did not differ between BIPAP+SB_mean _or PSV and controlled mechanical ventilation [11]. In contrast, Yoshida and colleagues [10] suggested that, in patients with ALI, improvement of lung aeration is more pronounced during BIPAP+SB_mean _than PSV. However, both in an animal [11] and patient study [10], aeration was assessed at end-expiration with static computed tomography (CT) during breath holding, possibly introducing artifacts. As dynamic CT (CT_dyn_) does not require breath holding, it may be considered a suitable technique for assessing lung aeration and ventilation during BIPAP+SB_mean _and PSV.

In the current study, we investigated the distributions of regional aeration and ventilation at the lungs' apex, hilum and base during PSV and BIPAP+SB_mean _using CT_dyn _in experimental ALI. We hypothesized that BIPAP+SB_mean_, compared with PSV: is associated with decreased amounts of nonaerated lung tissue and increased relative ventilation in dorsal lung zones due to increased inspiratory effort; and decreases tidal reaeration and hyperaeration through reduction of nonaerated lung tissue and different distribution of ventilation.

## Materials and methods

The protocol of this study has been approved by the local animal care committee and the Government of the State Saxony, Germany. Ten pigs (weighing 25.0 to 36.5 kg) were pre-medicated and anesthetized with intravenous midazolam, ketamine, and remifentanil. The trachea was intubated and lungs were ventilated with an EVITA XL 4 Lab (Dräger Medical AG, Lübeck, Germany) in the volume-controlled mode using a V_T _of 12 ml/kg, inspiratory: expiratory ratio (I:E) of 1:1, fraction of inspired oxygen (FiO_2_) of 0.5, positive end-expiratory pressure (PEEP) of 5 cmH_2_O, and respiratory rate (RR) set to achieve normocapnia. We decided to use a PEEP of 5 cmH_2_O to allow a better differentiation of tidal recruitment/reaeration and tidal hyperaeration between the modes investigated. Previous data from our group [12] suggest that such phenomena occur simultaneously but in different proportions depending on the level of PEEP. A FiO_2 _of 0.5 was chosen to allow adequate oxygenation without increasing atelectasis. FiO_2 _and PEEP were not changed during the experiments. An esophageal catheter (Erich Jaeger GmbH, Höchberg, Germany) was advanced through the mouth into the mid chest. A crystalloid solution (E153, Serumwerk Bernburg AG, Bernburg, Germany) at a rate of 10 to 20 mL.kg^-1^.h^-1 ^was used to maintain volemia.

Hemodynamics was monitored with catheters placed in right external carotid and pulmonary arteries. Arterial and mixed venous blood samples were analyzed.

Airway flow, airway pressure (P_aw_) and esophageal pressure were measured using calibrated flow and pressure sensors placed at the endotracheal tube, and respiratory parameters calculated. The ratio of inspiratory to total respiratory cycle (Ti/Ttot) was also determined. The product of inspiratory esophageal pressure vs. time (PTP), the difference between P_aw _at the beginning of inspiration and 100 ms thereafter (P_0.1_), and the dynamic intrinsic PEEP (PEEP_i,dyn_) were determined. Values of PTP, P_0.1 _and PEEP_i,dyn_ were taken from two minute and four minute recordings during controlled and assisted mechanical ventilation, respectively.

Respiratory parameters were computed from controlled (BIPAP+SB_controlled_) and spontaneous (BIPAP+SB_spont_) breath cycles. The contributions of spontaneous and controlled breaths to BIPAP+SB_mean _were weighted by their respective rates (weighted mean BIPAP+SB_mean_). Mean airway and transpulmonary pressures were weighted also by time, that is as the integral of the area under the flow curve divided by time, as shown in detail in Additional file 1.

### Dynamic computed tomography

CT_dyn _measurements were performed with a Somatom Sensation 16 (Siemens, Erlangen, Germany) at three different lung levels: apex (about 3 cm cranial to the carina); hilum (at carina level); base (about 2 to 3 cm caudal to the carina). Scans were obtained every 120 ms during a period of 60 seconds, resulting in approximately 500 images per level. Each image obtained corresponded to a matrix with 512 × 512 voxels of 0.443 × 0.443 × 1 mm^3^. Segmentation of the region of interest contained between the boundaries defined by the rib cage and mediastinal organs was performed semi-automatically, with software (CHRISTIAN II, Technical University Dresden, Germany) developed by one of the authors (MC). Each level was further divided into four zones of equal heights from ventral to dorsal (1 = ventral, 2 = mid-ventral, 3 = mid-dorsal, and 4 = dorsal). The four zones had equal height at each different level (apex, hilus, and base).

Aeration compartments at end-expiration and end-inspiration were computed based on an arbitrary scale for attenuation described elsewhere [13]. Accordingly, ranges of -1000 to -900 Hounsfield units (HU), -900 to -500 HU, -500 to -100 HU, and -100 to +100 HU were used to define the hyperaerated, normally aerated, poorly aerated, and nonaerated compartments, respectively.

Tidal reaeration was calculated as the decrease in the percentage of nonaerated and poorly aerated compartments from end-expiration to end-inspiration [14]. Tidal hyperaeration was calculated as the increase in the percentage of hyperaeration from end-expiration to end-inspiration [14].

Ventilation in one zone of a given level was computed as the variation of gas content between end-inspiration and end-expiration of that zone divided by the total variation of gas content in the respective level.

For BIPAP+SB_mean_, CT variables were computed in the same way as for respiratory parameters, that is weighted means of spontaneous and controlled breaths.

### Protocol for measurements

After preparation, animals were allowed to stabilize for 15 minutes (baseline, volume-controlled mode). ALI was induced by means of surfactant depletion [15] and considered stable if partial pressure of oxygen (PaO_2_)/FiO_2 _was 200 mmHg or less for at least 30 minutes (injury, volume-controlled mode). After obtaining the measurements at injury, BIPAP+SB_controlled _was initiated as follows: the driving pressure, which corresponded to the difference between the higher and the lower continuous positive P_aw _level of 5 cmH_2_O, was set to obtain V_T _of 7 to 8 ml/kg and mechanical RR was set to reach partial pressure of carbon dioxide (PaCO_2_) in the range of 50 to 60 mmHg, without spontaneous breathing. The I:E ratio was set to achieve mean P_aw _in the range of 8 to 10 cmH_2_O, as expected in PSV. At the same time, depth of anesthesia was a reduced, remaining constant thereafter. Lower mechanical RR combined with reduced depth of anesthesia enabled spontaneous breathing (unsynchronized and superimposed to BIPAP+SB_controlled_). When spontaneous breathing represented 20% or more of total minute ventilation, all animals were subjected to BIPAP+SB_mean _and PSV in randomized sequence for 60 minutes. During BIPAP+SB_mean_, the initial ventilatory settings of BIPAP+SB_controlled _were kept unchanged and spontaneous breathing efforts and rate increased according to the respiratory drive of the animals, without pressure support. During PSV, the target pressure support was set to achieve V_T _of 7 to 8 ml/kg, the inspiratory flow trigger was fixed at 2.0 L/min and the ventilator cycled-off at 25% of peak flow. Each assisted mechanical ventilation mode lasted 60 minutes. Measurements were performed at the following steps: baseline, injury and at the end of each assisted mechanical ventilation mode. The time elapsed between stabilization of injury, and first and second assisted mechanical ventilation mode corresponded to 60 and 120 minutes, respectively.

### Statistics

Data are given as mean ± standard deviation. Changes in functional variables were tested with two-tailed student's paired t-tests. Variables derived from CT_dyn _measurements were evaluated with mixed linear models using the following factors: level (apex, hilum, and base), zone (1 to 4) and type of mechanical ventilation (PSV, BIPAP+SB_mean_, BIPAP+SB_controlled _and BIPAP+SB_spont_). Compound symmetry for the measures on the same animal was assumed. Identical correlations were also assumed and their strength was estimated by components of variance. Residuals were checked for normal distribution, as suggested by their plots. Final mixed linear models resulted from stepwise model choices and included only statistical significant effects. Multiple comparisons were adjusted by the Bonferroni procedure. Univariate and multivariate analysis were performed with the software SPSS (Version 15.0, Chicago, IL, USA) and SAS (Procedure Mixed, Version 8, SAS Institute Inc, Cary, NC, USA), respectively. Statistical significance was accepted at *P *< 0.05 in all tests.

## Results

### Induction of acute lung injury

ALI was achieved with one to five lavages (median = 2.5), resulting in increased peak and mean P_aw _and mean transpulmonary pressure (Ppeak, Pmean, and Ppl mean, respectively; Table 1), as well as reduced oxygenation and increased mean pulmonary artery pressure (Table 2).

**Table 1:** Respiratory parameters

	Baseline	Injury	PSV	**BIPAP+SB**_**mean**_	**BIPAP+SB**_**controlled**_	**BIPAP+SB**_**spont**_
MV (L/min)	5.1 ± 1.7	4.1 ± 1.7	6.6 ± 1.9	6.2 ± 2.1	2.3 ± 0.9 ^†,‡^	4.0 ± 2.2 ^†,‡,§^
V_T _(mL)	347 ± 58	349 ± 61	202 ± 48	129 ± 40 ^†^	255 ± 103	97 ± 34 ^†,‡,§^
RR (/min)	15 ± 4	14 ± 4	34 ± 11	51 ± 17 ^†^	9 ± 3 ^†,‡^	43 ± 17 ^†,‡,§^
Ti/Ttot	0.49 ± 0.01	0.49 ± 0.01	0.33 ± 0.05	--	0.24 ± 0.08 ^†^	0.26 ± 0.06 ^†,‡^
Ppeak (cmH_2_O)	20 ± 2	34 ± 3 *	23 ± 2	--	24 ± 3	--
P_aw _mean (cmH_2_O)	11 ± 1	15 ± 1 *	9 ± 1	9 ± 1	14 ± 2 ^†,‡^	5 ± 1 ^†,‡,§^
Ppl mean (cmH_2_O)	3 ± 2	7 ± 2 *	2 ± 1	2 ± 1	6 ± 3 ^†,‡^	1 ± 1 ^†,‡,§^
PEEP_i,dyn _(cmH_2_O)	--	--	1 ± 1	1 ± 1	--	--
PTP (cmH_2_O.s.min^-1^)	--	--	7 ± 5	91 ± 54 ^†^	--	--
P0.1 (cmH_2_O)	--	--	1 ± 1	3 ± 1 ^†^	--	--

Values are given as mean ± standard deviation; baseline, before induction of acute lung injury; injury, after induction of acute lung injury. The contributions of spontaneous and controlled breaths to BIPAP+SB_mean _were weighted by their respective rates (weighted mean). * *P *< 0.05 vs. Baseline; ^† ^*P *< 0.05 vs. PSV; ^‡ ^*P *< 0.05 vs. BIPAP+SB_mean_; ^§ ^*P *< 0.05 vs. BIPAP+SB_contolled_.

BIPAP+SB_controlled_, controlled breath cycles during BIPAP+SB_mean_; BIPAP+SB_mean_, biphasic positive airway pressure + spontaneous breathing; BIPAP+SB_spont_, spontaneous breath cycles during BIPAP+SB_spont_; MV, minute volume; P_0.1_, airway pressure generated 100 ms after onset of an occluded inspiratory effort; P_aw _mean, mean airway pressure; PEEP_i,dyn_, dynamic intrinsic end-expiratory pressure; Ppeak, peak airway pressure; Ppl mean, mean transpulmonary pressure; PSV, pressure support ventilation; PTP, inspiratory esophageal pressure time product; RR, respiratory rate; Ti/Ttot, inspiratory to total respiratory time; V_T_, tidal volume.

**Table 2:** Gas exchange and hemodynamic variables

	Baseline	Injury	PSV	**BIPAP+SB**_**mean**_
**Gas exchange**				
PaO_2_/FIO_2_ (mmHg)	513 ± 62 (489.6-547.2)	119 ± 30* (92.0-143.1)	264 ± 127 (136.0-378.7)	246 ± 112 (143.1-332.5)
(%)	5.5 ± 1.4 (4.4-6.5)	33.9 ± 12.8* (24.7-39.7)	16.9 ± 10.4 (6.7-24.3)	19.8 ± 12.1 (11.6-28.7)
PaCO_2_ (mmHg)	34 ± 6 (29.4-39.7)	39 ± 8* (30.1-46.5)	48 ± 6 (44.2-55.2)	59 ± 13^†^ (46.9-66.2)

**Hemodynamics**				
CO (L/min)	3.2 ± 0.8 (2.4-3.8)	3 ± 0.8 (2.3-3.8)	4.3 ± 1.4 (2.8-5.3)	4.2 ± 1.2 (3.2-5.2)
HR (/min)	77 ± 13 (69-83)	75 ± 12 (65-86)	91 ± 18 (83-100)	91 ± 19 (77-110)
MAP (mmHg)	73 ± 9 (67-79)	69 ± 12 (62-75)	75 ± 8 (71-77)	79 ± 14 (68-98)
MPAP (mmHg)	22 ± 4 (20-24)	30 ± 5* (27-32)	31 ± 5 (26-35)	33 ± 6 (30-36)
CVP (mmHg)	10 ± 3 (8-12)	11 ± 2 (10-11)	9 ± 2 (8-10)	9 ± 2 (7-11)
PCWP (mmHg)	13 ± 2 (12-14)	14 ± 2 (12-15)	13 ± 4 (11-14)	12 ± 2 (11-15)

Values are given as mean ± standard deviation. Baseline, before induction of acute lung injury; injury, after induction of acute lung injury. * *P *< 0.05 vs. baseline; _† _*P *< 0.05 vs. PSV.

BIPAP+SB_mean_, biphasic positive airway pressure + spontaneous breathing; CO, cardiac output; CVP, central venous pressure; FiO_2_, fraction of inspired oxygen; HR, heart rate; MAP, mean arterial pressure; MPAP, mean pulmonary arterial pressure; PaCO_2_, partial pressure of arterial carbon dioxide; PaO_2_, partial pressure of arterial oxygen; PCWP, pulmonary artery occlusion pressure; PSV, pressure support ventilation; , mixed venous admixture.

### Assisted mechanical ventilation

During BIPAP+SB_mean _we detected spontaneous breathing only on low but not on high continuous positive P_aw _levels. Minute ventilation did not differ between PSV and BIPAP+SB_mean _(Table 1). However, mean V_T _was higher, whereas mean RR was lower during PSV. Ppeak during BIPAP+SB_controlled _and PSV were comparable. The time spent during inspiration was proportionally shorter in BIPAP+SB_mean _than PSV, as reflected by Ti/Tot. Pmean during BIPAP+SB_mean _did not differ from PSV. However, Pmean and Ppl mean were higher during BIPAP+SB_controlled _and lower during BIPAP+SB_spont _as compared with PSV. PEEP_i,dyn _values did not differ between assisted mechanical ventilation modes, but values of P_0.1 _and PTP were higher during BIPAP+SB_mean _compared with PSV.

Arterial oxygenation and hemodynamic variables did not differ between the assisted mechanical ventilation modes, but PaCO_2 _was higher during BIPAP+SB_mean _than PSV (Table 2).

The statistical analysis evidenced no effect of the sequence of ventilation modes on the hyperaerated, normally aerated, poorly aerated, and nonaerated compartments at end-expiration. The Additional files 2 and 3 show CT_dyn _videos of lungs during BIPAP+SB_mean _and PSV in one animal, respectively.

**Figure 1:**
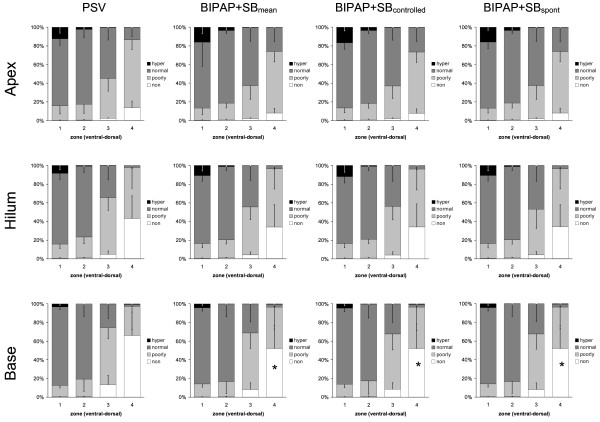
**Distributions of hyperaerated (hyper), normally aerated (normal), poorly aerated (poorly) and nonaerated (non) compartments at end-expiration during pressure support ventilation (PSV), biphasic positive pressure ventilation + spontaneous breaths (BIPAP+SB_mean_), controlled (BIPAP+SB_controlled_) and spontaneous (BIPAP+SB_spont_) breath cycles**. Calculations were performed for different lung zones from ventral to dorsal (1 = ventral, 2 = mid-ventral, 3 = mid-dorsal, and 4 = dorsal) at lungs apex, hilum, and base using dynamic computed tomography. The contributions of BIPAP+SB_spont _and BIPAP+SB_controlled _to BIPAP+SB_mean _were weighted by their respective rates (weighted mean). Bars and vertical lines represent means and standard deviations, respectively. * *P *< 0.05 vs. PSV; † *P *< 0.05 vs. BIPAP+SB_controlled_.

**Figure 2:**
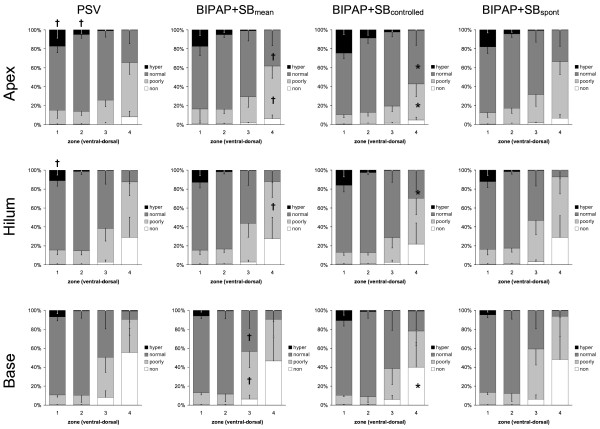
**Distributions of hyperaerated (hyper), normally aerated (normal), poorly aerated (poorly) and nonaerated (non) compartments at end-inspiration during pressure support ventilation (PSV), biphasic positive pressure ventilation + spontaneous breaths (BIPAP+SB_mean_), controlled (BIPAP+SB_controlled_) and spontaneous (BIPAP+SB_spont_) breath cycles**. Calculations were performed for different lung zones from ventral to dorsal (1 = ventral, 2 = mid-ventral, 3 = mid-dorsal, and 4 = dorsal) at lungs apex, hilum, and base using dynamic computed tomography. The contributions of BIPAP+SB_spont _and BIPAP+SB_controlled _to BIPAP+SB_mean _were weighted by their respective rates (weighted mean). Bars and vertical lines represent means and standard deviations, respectively. * *P *< 0.05 vs. PSV; † *P *< 0.05 vs. BIPAP+SB_controlled_.

During BIPAP+SB_mean _and PSV, we observed at end-expiration and end-inspiration (Figures 1 and 2, respectively) a gravity-dependent loss of lung aeration, characterized by increase of nonaerated and poorly aerated areas, as well as decrease in hyperaerated and normally aerated tissue in dorsal zones, as compared with ventral ones (*P *< 0.0001). Similarly, the percentages of nonaerated and poorly aerated areas increased, whereas those from normally aerated and hyperaerated areas decreased from lung apex to base following the gravitational gradient, independent from the assisted mechanical ventilation mode and lung zone (*P *< 0.0001).

Compared with PSV, BIPAP+SB_controlled _and BIPAP+SB_spont _resulted in a reduction of the percentage of nonaeration at end-expiration at the lung base (Figure 1, *P *< 0.05). At end-inspiration, BIPAP+SB_mean _led to an increased percentage of normally aerated tissue at apex and hilum, as well as reduced poorly aerated and nonaerated tissue at apex and base, respectively, mainly during controlled breaths (Figure 2, *P *< 0.05). The distribution of aeration during BIPAP+SB_controlled _and BIPAP+SB_spont _was comparable at end-expiration, as well as end-inspiration (Figures 1 and 2, respectively).

**Figure 3:**
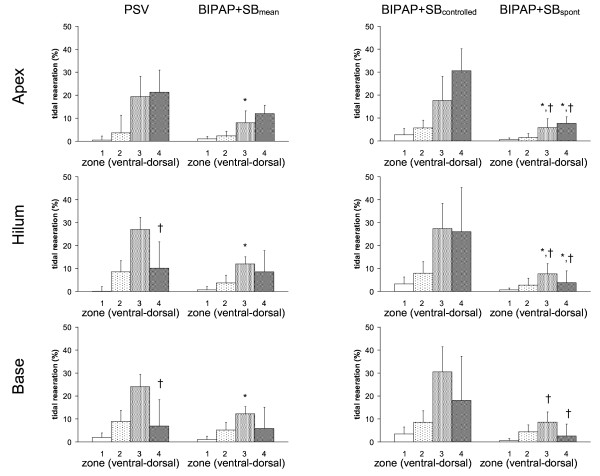
**Tidal reaeration during pressure support ventilation (PSV), biphasic positive pressure ventilation + spontaneous breaths (BIPAP+SB_mean_), controlled (BIPAP+SB_controlled_) and spontaneous (BIPAP+SB_spont_) breath cycles**. Calculations were performed for different lung zones from ventral to dorsal (1 = ventral, 2 = mid-ventral, 3 = mid-dorsal, and 4 = dorsal) at lungs apex, hilum, and base using dynamic computed tomography. The contributions of BIPAP+SB_spont _and BIPAP+SB_controlled _to BIPAP+SB_mean _were weighted by their respective rates (weighted mean). Bars and vertical lines represent means and standard deviations, respectively. * *P *< 0.05 vs. PSV; † *P *< 0.05 vs. BIPAP+SB_controlled_.

As shown in Figure 3, tidal reaeration had a gravity-dependent pattern (*P *< 0.0001), increasing from ventral to mid-dorsal (*P *< 0.0001), but decreasing from mid-dorsal to dorsal zones (*P *< 0.0001). Compared with PSV, BIPAP+SB_mean _induced less tidal reaeration in mid-dorsal zones, mainly due to spontaneous breaths. Also, in dorsal zones, tidal reaeration was more pronounced during PSV than BIPAP+SB_spont_. On the other hand, tidal reaeration was less marked during PSV than controlled breaths of BIPAP+SB_mean_.

**Figure 4:**
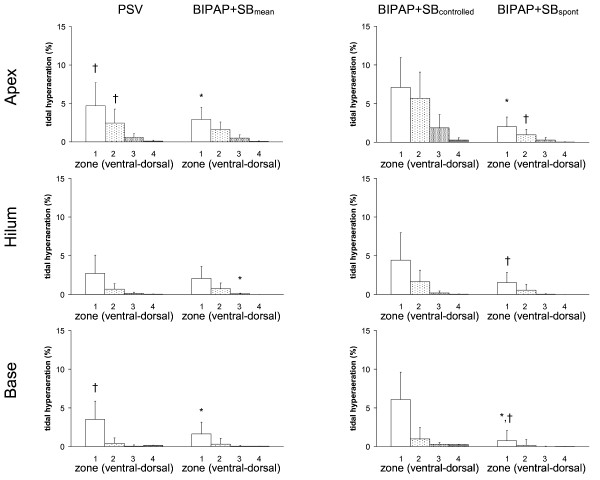
**Tidal hyperaeration during pressure support ventilation (PSV), biphasic positive pressure ventilation + spontaneous breaths (BIPAP+SB_mean_), controlled (BIPAP+SB_controlled_) and spontaneous (BIPAP+SB_spont_) breath cycles**. Calculations were performed for different lung zones from ventral to dorsal (1 = ventral, 2 = mid-ventral, 3 = mid-dorsal, and 4 = dorsal) at lungs apex, hilum, and base using dynamic computed tomography. The contributions of BIPAP+SB_spont _and BIPAP+SB_controlled _to BIPAP+SB_mean _were weighted by their respective rates (weighted mean). Bars and vertical lines represent means and standard deviations, respectively. * *P *< 0.05 vs. PSV; † *P *< 0.05 vs. BIPAP+SB_controlled_.

Tidal hyperaeration increased from dorsal to ventral lung zones, as well as from apex to base (Figure 4, *P *< 0.0001 both). Tidal hyperaeration was decreased during BIPAP+SB_mean _compared with PSV. In ventral zones of the lung apex and base, tidal hyperaeration increased during controlled but decreased during BIPAP+SB_spont _compared with PSV.

Distribution of ventilation did not differ among the lung levels, but was lowest in ventral and highest in mid-ventral zones (*P *< 0.0001 both). No differences were observed among PSV, BIPAP+SB_mean_, BIPAP+SB_controlled _and BIPAP+SB_spont _(*P *= 1.0).

## Discussion

In a surfactant depletion model of ALI, we found that BIPAP+SB_mean _compared with PSV resulted in: lower mean V_T_, comparable oxygenation and hemodynamics, and increased PaCO_2 _and inspiratory effort; less nonaerated areas at end-expiration; decreased tidal hyperaeration and reaeration; and similar distributions of relative ventilation. During BIPAP+SB_mean_: BIPAP+SB_spont _had lower V_T _and higher rate than BIPAP+SB_controlled_; BIPAP+SB_spont _and BIPAP+SB_controlled _had similar distributions of ventilation and aeration; BIPAP+SB_controlled _resulted in increased tidal reaeration and hyperareation, compared with PSV. BIPAP+SB_spont _showed an opposite pattern.

To our knowledge, this is the first study showing that despite reduced nonaerated lung tissue during BIPAP+SB_mean _compared with PSV, differences in tidal reaeration and hyperaeration seem to be due only to lower V_T _of spontaneous breaths, because the distribution ventilation are comparable.

The present study differs from previous investigations on BIPAP+SB_mean _and PSV [6,9-11] in that: CT_dyn _was used to assess regional aeration during up to 60 seconds; no breath holds at end-expiration or end-inspiration were used; and both the mean P_aw _and minute ventilation were comparable between BIPAP+SB_mean _and PSV. Different investigators have used CT_dyn _to quantify lung aeration, detect tidal recruitment and derecruitment, as well hyperaeration in ALI/acute respiratory distress syndrome (ARDS) [8,16,17]. When negative intrapleural pressures are generated, CT_dyn _seems to be superior to static helical CT for quantifying lung aeration at mid-expiration and mid-inspiration [18]. Furthermore, as V_T _during BIPAP+SB_mean _and PSV are not constant [19], aeration measurements taken within a single breath may be less representative of longer periods of ventilation.

### Aeration compartments

Compared with PSV, BIPAP+SB_mean _reduced the percentage of nonaerated areas at end-expiration in dependent lung zones, both BIPAP+SB_controlled _and BIPAP+SB_spont_. At end-inspiration, the patterns of distribution of aeration were similar between BIPAP+SB_mean _and PSV. Nonetheless, BIPAP+SB_controlled _showed less poorly aerated and more normally aerated percentages of lung tissue than BIPAP+SB_mean_. Two mechanisms can explain these observations. First, spontaneous breathing may have favored recruitment of more dependent zones at end-expiration, with effects being preserved during controlled breaths. This hypothesis is supported by increased PTP and Ppl mean during BIPAP+SB_mean _compared with PSV. Second, BIPAP+SB_controlled _generated higher products of P_aw _in time during inspiration, as shown by our data, thus promoting recruitment of lung zones with increased time constants, with effects being preserved during BIPAP+SB_spont_. Indeed, it has been shown that in controlled ventilation the more tissue is recruited at end-inspiration, the more tissue remains recruited at end-expiration [20]. On the other hand, the amount of hyperaeration at end-inspiration was higher during BIPAP+SB_controlled _than PSV, despite comparable Ppeak. The most probable explanation is that Pmean was higher during BIPAP+SB_controlled _than PSV. Another likely explanation is that the gas volume at end-expiration was higher, as suggested by lower percentages of nonaerated areas during BIPAP+SB_mean_, generating an overall shift towards more aeration. Accordingly, hyperaeration was more localized in non-dependent lung zones. However, mean hyperaeration at end-inspiration was comparable between BIPAP+SB_mean _and PSV, due to less hyperaeration during BIPAP+SB_spont_.

### Tidal reaeration and hyperaeration

Tidal recruitment or reaeration and tidal hyperaeration have been proposed to reflect the phenomena of cyclic collapse/reopening and overdistension of lung units in ALI/ARDS [14,21], which are important risk factors for ventilator-associated lung injury [22]. Recruitment occurs mainly in nonaerated tissue [21], but seems to also take place in the poorly aerated tissue [14]. Tidal reaeration and hyperaeration have been described during studies on controlled mechanical ventilation [14,21,23,24], but data during assisted mechanical ventilation are scarce. Wrigge and colleagues [8] reported in an oleic acid model of ALI, more aeration and less tidal recruitment in dependent lung zones during BIPAP+SB_mean _compared with pressure-controlled ventilation. However, other forms of assisted mechanical ventilation were not addressed. We found that mean tidal hyperaeration and reaeration were less pronounced during BIPAP+SB than PSV. However, when analyzed separately, we found that BIPAP+SB_controlled _were associated with increased tidal hyperaeration and reaeration compared with PSV, whereas BIPAP+SB_spont _showed the opposite pattern. As mean V_T _and Ppl were lower during BIPAP+SB_spont _than BIPAP+SB_controlled_, BIPAP+SB_mean _could be claimed to be more lung protective than PSV due to lower mean distending volumes/pressures during spontaneous breathing. On the other hand, Plpl, tidal hyperaeration and reaeration were more pronounced during BIPAP+SB_controlled _than PSV. Thus, the phenomena of cyclic collapse-reopening and overdistension may be more significant if the proportion of controlled to spontaneous breaths during BIPAP+SB_mean _is high. Furthermore, RR was higher during BIPAP+SB_mean _compared with PSV, which may favor lung injury [25]. Our findings raise the question on how much spontaneous breathing should be allowed or used during BIPAP+SB_mean _to improve respiratory function and reduce ventilator-associated lung injury. However, it was beyond the scope of this work to determine the impact of BIPAP+SB_mean _and PSV on lung injury.

### Distribution of ventilation and gas exchange

As BIPAP+SB_mean _was associated with increased inspiratory effort, we expected the relative ventilation to be higher with that mode in the most dependent lung zones compared with PSV [26]. However, the distribution of ventilation was similar during BIPAP+SB_mean _and PSV, both during spontaneous and controlled breaths. The most likely explanation is that although the inspiratory transpulmonary pressures in dependent zones increased aeration during BIPAP+SB_mean _compared with PSV, the impedance to ventilation was likely to not be changed and shift of relative ventilation did not occur.

As the percentage of nonaerated areas was decreased during BIPAP+SB_mean _compared with PSV, we expected an improvement in oxygenation. However PaO_2_/FIO_2 _and venous admixture were comparable between modes, suggesting that hypoxic vasoconstriction most likely played a role. BIPAP+SB_mean _results in increased redistribution of pulmonary blood flow from dorsal to ventral zones [11]. Two possible mechanisms may explain limited carbon dioxide exchange during BIPAP+SB_mean _compared with PSV, despite similar minute ventilation. First, total alveolar ventilation was reduced due low V_T _in spontaneous breaths. Second, during controlled breaths, higher dead space due to increased hyperaerated areas may have occurred.

### Limitations

This study has several limitations. First, the surfactant depletion model does not reproduce all features of clinical ALI and extrapolation of our results to the clinical scenario is limited. Second, artifacts introduced by the cranial-caudal movement of lungs were not compensated during calculations of aeration by CT_dyn_, and levels chosen for the slices may have slightly differed between ventilation modes. However, measurements were performed at three different lung levels and we did observe regional differences. Furthermore, the levels used for CT scans were referred to anatomical landmarks (carina), likely reducing such artifacts. Third, tidal aeration and hyperaeration calculations were of volumetric nature. As hyperaerated areas have proportionally low mass, the absolute amount of lung tissue undergoing cyclic hyperaeration may be reduced. On the other hand, the thresholds for CT compartments most likely resulted in underestimation of hyperaeration in ALI, but they correspond to those internationally recommended [27,28]. Fourth, the assessment of relative ventilation by changes in CT densities may have been skewed by movement of gas within structures with limited participation in gas exchange, like small airways. Nevertheless, stress/strain of those structures seems to play an important role in ventilator-induced lung injury [29]. Fifth, we did not determine the impact of BIPAP+SB_mean _and PSV on lung mechanical stress and inflammation directly. However, in experimental ALI, tidal hyperaeration and reaeration seem to be closely related to overdistension and collapse/reopening of lung units, respectively [12,14,30].

## Conclusions

In this model of ALI, the reduction of tidal reaeration and hyperaeration during BIPAP+SB_mean _compared with PSV is not due to decreased nonaerated areas at end-expiration or different distribution of ventilation, but to lower V_T _during BIPAP+SB_spont_.

## Key messages

• Compared with PSV, BIPAP+SB_mean _resulted in: lower mean V_T_, comparable oxygenation and hemodynamics, and increased PaCO_2 _and inspiratory effort; less nonaerated areas at end-expiration; decreased tidal hyperaeration and reaeration; similar distributions of relative ventilation.

• During BIPAP+SB_mean_: BIPAP+SB_spont _had lower V_T _and higher rate than BIPAP+SB_controlled_; BIPAP+SB_spont _and BIPAP+SB_controlled _had similar distributions of ventilation and aeration; BIPAP+SB_controlled _resulted in increased tidal reaeration and hyperareation, compared with PSV, while BIPAP+SB_spont _showed an opposite pattern.

• The ratio between spontaneous to controlled breaths could play an important role in reducing tidal reaeration and hyperaeration during BIPAP+SB_mean_.

## Abbreviations

ALI: acute lung injury; ARDS: acute respiratory distress syndrome; BIPAP+SB_controlled_: time-cycled controlled breaths at two levels of continuous positive airway pressure during BIPAP+SB_mean_; BIPAP+SB_mean_: biphasic positive airway pressure with non-assisted spontaneous breathing; BIPAP+SB_spont_: non-assisted spontaneous breathing during BIPAP+SB_mean_; CT: computed tomography; CT_dyn_: dynamic computed tomography; FiO_2_: fraction of inspired oxygen; HU: Hounsfield units; I:E: inspiratory:expiratory ratio; P_0.1_: decay in airway pressure 100 ms after begin of the inspiration; PaCO_2_: partial pressure of arterial carbon dioxide; PaO_2_: partial pressure of arterial oxygen; P_aw_: airway pressure; PEEP: positive end-expiratory pressure; PEEP_i,dyn_: dynamic intrinsic end-expiratory pressure; Pmean: mean airway pressure; Ppeak: peak airway pressure; Ppl mean: mean transpulmonary pressure; PSV: pressure support ventilation; PTP: pressure versus time product of the inspiratory esophageal pressure; RR: respiratory rate; Ti/Ttot: inspiratory to total respiratory time; V_T_: tidal volume.

## Competing interests

The authors declare that they have no competing interests.

## Authors' contributions

All authors made substantial contribution to the study design. MGA and MC drafted the manuscript and helped to perform the experiments. PM, ARC and CS helped to perform the experiments and contributed to drafting the manuscript. MC and VH developed the software for analysis of dynamic computed tomography scans and helped to draft the manuscript. BW performed the more complex multivariate statistical analysis and helped to draft the manuscript. All other authors revised the manuscript for important intellectual content. All authors approved the final version of the manuscript for publication.

## Supplementary Material

Additional file 1 **Calculation of mean airway pressures**. This file shows exactly how the mean airway pressures were calculated for the different modes of assisted ventilation, including the spontaneous and controlled cycles of biphasic positive airway pressure + spontaneous breathing (BIPAP+SB_mean_). Click here for file

Additional file 2 **Dynamic computed tomography in a representative animal during biphasic positive airway pressure + spontaneous breathing (BIPAP+SB_mean_)**. This video shows a dynamic computed tomography scan (grey scale) of the chest taken for approximately 60 seconds at the hilus in one representative animal during assisted ventilation with BIPAP+SB_mean_. Acute lung injury was induced by surfactant depletion. See Additional file 3 for comparison with pressure support ventilation (PSV). Click here for file

Additional file 3 **Dynamic computed tomography in a representative animal during pressure support ventilation (PSV)**. This video shows a dynamic computed tomography scan (grey scale) of the chest taken for approximately 60 seconds at the hilus in a representative animal during assisted ventilation with PSV. Acute lung injury was induced by surfactant depletion. See Additional file 2 for comparison with biphasic positive airway pressure + spontaneous breathing (BIPAP+SB_mean_). Click here for file
