# Supplementary material for: Regional lung aeration and ventilation during pressure support and biphasic positive airway pressure ventilation in experimental lung injury
Source: Crit Care. 2010 Mar 16;14(2):R34. doi: 10.1186/cc8912 (PMC2887141; doi:10.1186/cc8912)
Supplement: Additional file 1 — Calculation of mean airway pressures. This file shows exactly how the mean airway pressures were calculated for the different modes of assisted ventilation, including the spontaneous and controlled cycles of biphasic positive airway pressure + spontaneous breathing (BIPAP+SBmean). [file cc8912-S1.DOC]

*Mean airway pressures were calculated taking the time into account. In this sense, the term “weighed” considers not only the number of breaths, but also the time a given pressure has been applied, as follows:*

$Paw\_mean=\int_{begin}^{end} Paw(t)\cdot dt$ *(1)*

*Equation 1 was used to calculate the Paw mean of BIPAP+SBmean.*

*Accordingly, calculations of Paw for spontaneous and controlled, or mandatory, breath cycles of BIPAP must take the duration of respective breaths into account, as follows:*

$Paw\_BIPAP+SBspont=\frac{\sum_{i=1}^{n\_s} \int_{begin\_of\_cycle\_i}^{end\_of\_cycle\_i} Paw_{i}(t)\cdot dt}{n\_s}$ *(2)*

*In Equation 2, i represents the number of a given spontaneous cycle and n the total number of spontaneous cycles.*

*Calculations of Paw mean for controlled breaths of BIPAP was performed similarly, as follows:*

$Paw\_BIPAP+SBcontr=\frac{\sum_{i=1}^{n\_c} \int_{begin\_of\_cycle\_i}^{end\_of\_cycle\_i} Paw_{i}(t)\cdot dt}{n\_c}$ *(3)*

*Where n_c represents the total number of controlled breath cycles.*
